# Efficacy of Modified Bioactive Glass for Dentin Remineralization and Obstruction of Dentinal Tubules

**Published:** 2017-07

**Authors:** Mahshid Saffarpour, Maryam Mohammadi, Mohammadreza Tahriri, Azadeh Zakerzadeh

**Affiliations:** 1 Assistant Professor, Department of Operative Dentistry, School of Dentistry, Alborz University of Medical Sciences, Karaj, Iran; 2 Assistant Professor, Dental Research Center, School of Dentistry, Marquette University, Milwaukee, WI, USA; 3 Assistant Professor, Department of Operative Dentistry, School of Dentistry, Qazvin University of Medical Sciences, Qazvin, Iran

**Keywords:** Bioactive Glass, Strontium, Dentin, Tooth Remineralization

## Abstract

**Objectives:**

This study assessed the efficacy of modified bioactive glass (MBG) for dentin remineralization and obstruction of dentinal tubules.

**Materials and Methods:**

Thirty-six dentin discs were made from 20 third molars and were stored in 12% lactic acid solution for two weeks to induce demineralization. The samples were divided into three groups (n=12): 1- BG, 2- BG modified with 5% strontium (Sr) and 3- BG modified with 10% Sr. After applying the BG, the samples were stored in artificial saliva for 7, 14 and 21 days. Attenuated Total Reflection-Fourier Transform Infrared Spectroscopy (ATR-FTIR), X-ray Diffraction (XRD) analysis, Scanning Electron Microscopy (SEM), and Energy-Dispersive X-ray (EDX) analysis were used to assess remineralization. Also, 6 dentin discs were divided into three groups of BG, BG modified with 5% Sr and BG modified with 10% Sr, to examine tubular occlusion. The discs were etched using 0.5M of EDTA for two minutes and were stored in artificial saliva for 7 days. Changes in dentin surface morphology were evaluated under SEM.

**Results:**

Group 3 showed high rates of remineralization at days 7 and 14, although the rate decreased at day 21. Group 2 exhibited high rates of remineralization at days 7, 14 and 21. Dentinal tubules were partially occluded by BG and BG modified with 5% Sr, while they were almost completely obstructed after the use of BG modified with 10% Sr.

**Conclusions:**

Strontium increases remineralization. Addition of 10% Sr to BG enhances apatite formation; however, the apatite dissolves over time. Addition of 5% Sr to BG stabilizes the apatite lattice and increases the remineralization.

## INTRODUCTION

Tooth decay is a common cause of tooth loss [1]. Dental caries, once believed to be an irreversible bacterial infection, is a complex multifactorial disease. It develops as a result of a disparity between the dental mineral contents and biofilm. Dental caries is believed to be an active process with longer episodes of demineralization than remineralization [2]. This new concept has emphasized the importance of remineralizing incipient carious lesions [3].

The demineralization process can be halted by providing a suitable environment for remineralization through using different remineralizing agents [4]. If demineralization exceeds the self-regulated remineralization [5], excessive loss of mineral content may take place, endangering the integrity of the tooth [6]. Numerous studies have been conducted aiming at reincorporating minerals into demineralized dentin to achieve remineralization [7, 8]. Bioactive glass (BG) is a unique biomaterial, which has been extensively studied in the fields of tissue engineering, bone regeneration, and dentin remineralization, because of its remarkable bioactive capability in forming hydroxyl carbonate apatite (HCA) when stored in simulated body fluids. BG has novel properties, and plays a role as a biomimetic mineralizer, mirroring the inherent mineralization mechanisms that take place in vivo, while simultaneously affecting cell signals to benefit from the restoration of tissues and reestablishment of their function [9]. BG is made of sodium, calcium, phosphorous and silica (calcium sodium phosphosilicate), which are naturally found in human body. When in contact with saliva or water, BG particles release sodium, calcium and phosphorous ions into saliva, and remineralize dental surfaces [3]. Strontium (Sr) can substitute calcium in the BG composition. Sr has chemical and physical properties similar to those of calcium; thus, theoretically, it is capable of replacing calcium in the hydroxyapatite structure [10,11]. Recent studies have shown that Sr-substituted BG is favorable for bone repair and regenerative treatments [12]. Additionally, when Sr substitutes calcium, the glass network expands due to the larger ionic radius of Sr [13]. Dentin hypersensitivity (DH) is common in the clinical setting. Although the etiology of DH is multifactorial, the currently accepted theory for DH is the hydrodynamic theory, which proposes that when external stimuli, such as cold or heat are applied to exposed dentin, they cause fluid movement within dentinal tubules [14]. DH is managed using potassium oxalate, ferric oxalate and resins to occlude dentinal tubules. The desensitizing effect of these products is weak and short-term. On the other hand, BG occludes dentinal tubules with a calcium phosphate precipitate, which is similar to the major inorganic dentin component [6]. The current study was designed to compare the apatite-forming capacity of BG and Sr-modified BG in dentin remineralization using Attenuated Total Reflection-Fourier Transform Infrared Spectroscopy (ATR-FTIR). Qualitative analyses were performed using X-ray Diffraction (XRD) analysis and Scanning Electron Microscopy-Energy-Dispersive X-ray analysis (SEM-EDX), while tubular obstruction was evaluated using SEM.

## MATERIALS AND METHODS

### Preparation of samples:

Twenty extracted caries-free human third molars were obtained according to the protocol approved by the ethical code IR.QUMS.1394.387 of Qazvin University of Medical Sciences. The teeth were cleaned using hand instruments to remove soft tissue debris and were stored in 0.5% thymol solution. Dentin discs with the thickness of 1.0±0.1 mm were prepared by sectioning each tooth at 1.5mm above the cementoenamel junction (CEJ), using a low-speed water-cooled diamond disc (IsoMet, Buehler, Lake Bluff, IL, USA). The surrounding enamel was removed, and square-shaped dentin specimens with the dimensions of 5×5×1mm^3^ were formed.

### Tooth demineralization and treatment procedures:

The teeth were immersed in jars containing a 0.1M solution of 12% lactic acid for two weeks at 25°C to achieve the pH of 4. The acidified solution was refreshed every five days, and a pH-meter was used to ensure the pH did not change [14]. The specimens were monitored using digital radiography to ensure that the samples were demineralized [15].

After demineralization, the dentin discs were rinsed with distilled water for 2 minutes. The specimens were randomly divided into three groups (n=12). Three remineralizing agents were used: BG, BG modified with 5% Sr and BG modified with 10% Sr. The compositions of BG and modified BG are listed in Table 1. The dentin surfaces were slightly rubbed with 20mg of BG or modified BG using a wet cotton pellet for one minute, followed by copious rinsing with distilled water for one minute [9]. Then, the samples were stored in artificial saliva with the pH of 7.4 for 7, 14 and 21 days at 37°C to remineralize.

**Table 1.** Composition of the materials used for dentin remineralization

**Material**	**Composition**	**Particle size range (μm)**
BG	64% SiO_2_, 26% CaO, 10% P_2_O_5_	40–90
BG modified with 5% Sr	64% SiO_2_, 21% CaO, 10% P_2_O_5_, 5% Sr	10–90
BG modified with 10% Sr	64% SiO_2_, 16% CaO, 10% P_2_O_5_, 10% Sr	10–90

BG=Bioactive Glass, Sr=Strontium

The artificial saliva was composed of 0.86g sodium chloride (NaCl), 0.30g potassium chloride (KCl), and 0.33g calcium chloride (CaCl_2_) and di-H_2_O, and was refreshed every 24 hours.

### ATR-FTIR spectroscopy:

The ATR-FTIR spectra were obtained from the dentin specimens after 7, 14 and 21 days of remineralization in artificial saliva, using a Nicolet™ iS™ 10 FT-IR spectrometer (Thermo Fisher Scientific, Waltham, MA, USA). Potassium bromide (KBr) technique has been applied in the current study. The spectra were collected in the range of 350–4000 cm^−1^ at 10.4 cm^−1^ resolution for a total of 72 scans and were analyzed using OMNIC 8 software (Thermo Fisher Scientific, Waltham, MA, USA). Prior to spectrophotometric analysis, the specimens were rinsed with distilled water for 30 seconds and were completely air-dried. Additionally, ATR-FTIR spectra of BG powders were measured to identify their chemical structure.

### XRD analysis:

XRD analysis was performed at 7, 14 and 21 days, using X’Pert PRO XRD system (Bureau Veritas, Matraville, Australia), with a CuKα generator working at 40 kilovoltages (kV) and 40 milliamperes (mA). The diffraction intensity as a function of the angle 2-theta was measured between 20° and 55°, with a 2-theta step of 0.039°. An untreated dentin disc was also measured to acquire the spectrum of sound dentin. BG powders were also tested as control samples.

### SEM-EDX analysis:

Six demineralized specimens were remineralized for 7, 14 and 21 days, rinsed with distilled water, dried in a desiccator, and gold coated. Changes in dentin surface morphology were evaluated under SEM (XL30, Philips, IL, USA) at 20 kV. An EDX apparatus connected to the SEM was used to measure the content of the chemical elements in the samples.

### Preparation of teeth for analysis of tubular obstruction:

Six dentin discs were randomly divided into three groups (n=2): BG, BG modified with 5% Sr and BG modified with 10% Sr. The dentin discs were etched using 0.5M Ethylenediaminetetraacetic acid (EDTA) for two minutes. The dentin surfaces were slightly rubbed with 20mg of BG or modified BG using a wet cotton pellet for one minute and were rinsed with distilled water. Afterwards, the samples were stored in artificial saliva with the pH of 7.4 at 37°C for 7 days. The Six demineralized specimens were rinsed with distilled water, dried in a desiccator, and gold coated. Changes in dentin surface morphology were evaluated under SEM at 20 kV.

## RESULTS

### ATR-FTIR spectroscopy:

**Fig. 1:**
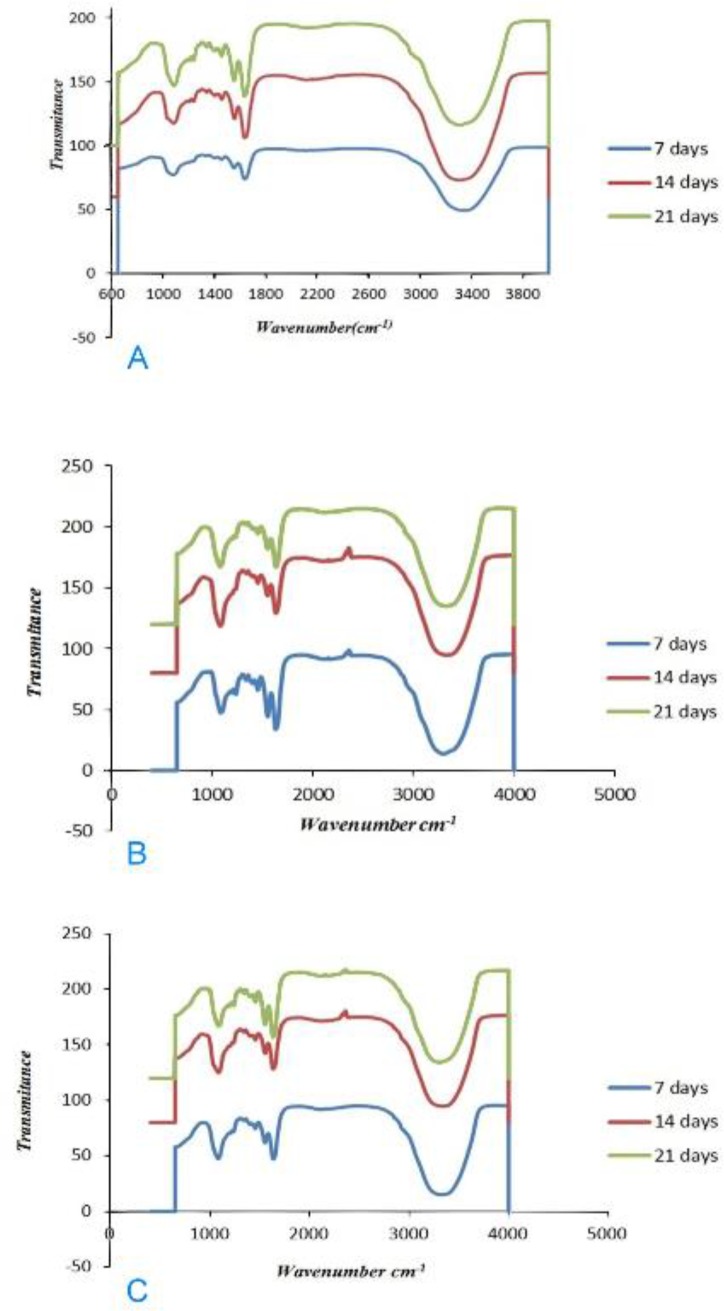
(A) ATR-FTIR of BG. (B) ATR-FTIR of BG modified with 5% Sr. (C). ATR-FTIR of BG modified with 10% Sr

All the representative spectra recorded in the region of 800–1800 cm^−1^ showed phosphate bands representative of mineral components. The phosphate bands increased during the seven-day period in the BG group. Increased phosphate peaks were also observed in the BG group after 14 and 21 days. The phosphate bands at 800–1800 cm^−1^ in the group of BG modified with 5% Sr increased significantly over time, indicating that remineralization after 21 days was greater than that after 7 and 14 days.The phosphate bands at 800–1800 cm^−1^ in the group of BG modified with10% Sr after 7 and 14 days had greater intensity when compared to those in the groups of BG and BG modified with 5% Sr; however, the intensity of the bands decreased after 21 days of remineralization. The ATR-FTIR analyses performed on demineralized dentin after 7, 14 and 21 days of remineralization are displayed in Figure 1 (A–C).

### XRD measurement:

**Fig. 2:**
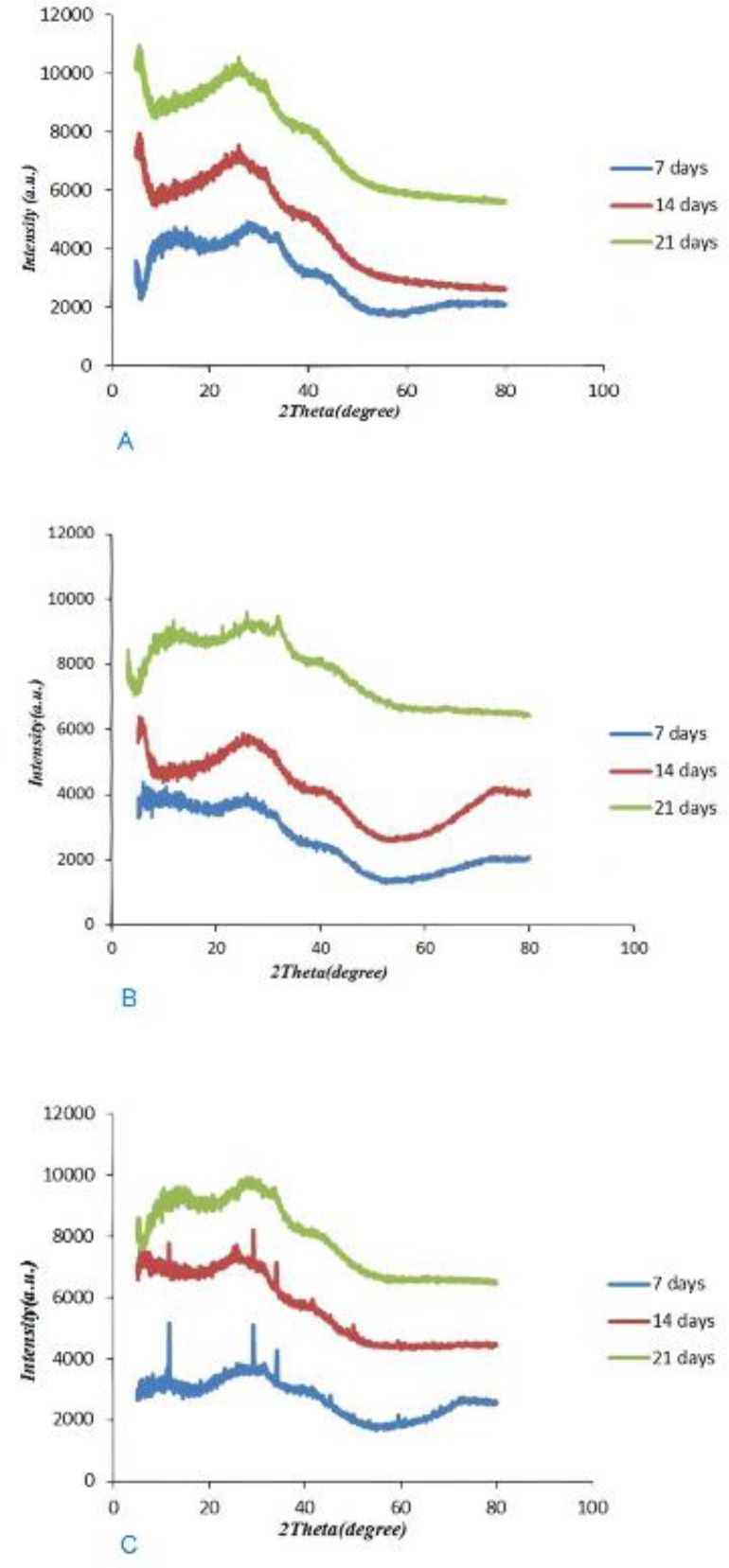
(A) XRD of BG. (B) XRD of BG modified with 5% Sr. (C) XRD of BG modified with 10% Sr

The results of XRD analysis after 7, 14 and 21 days of remineralization following treatments with the three agents are shown in Figure 2 (A–C). After 7 days of remineralization, the diffraction patterns exhibited a peak at 2θ=20–35°; however, sharp diffraction peaks or noisy peaks were not seen in the BG group. After 14 and 21 days of remineralization, the diffraction patterns exhibited a more obvious halo at 2θ=20–35°. In the group of BG modified with 5% Sr, the diffraction patterns at 2θ=20–35° showed more obvious peaks after 21 days, when compared to days 7 and 14. The diffraction patterns at 2θ=20–35° exhibited sharp peaks with a high intensity in the group of BG modified with 10% Sr at days 7 and 14; however, the peak decreased after 21 days.

### SEM-EDX analysis:

**Fig. 3:**
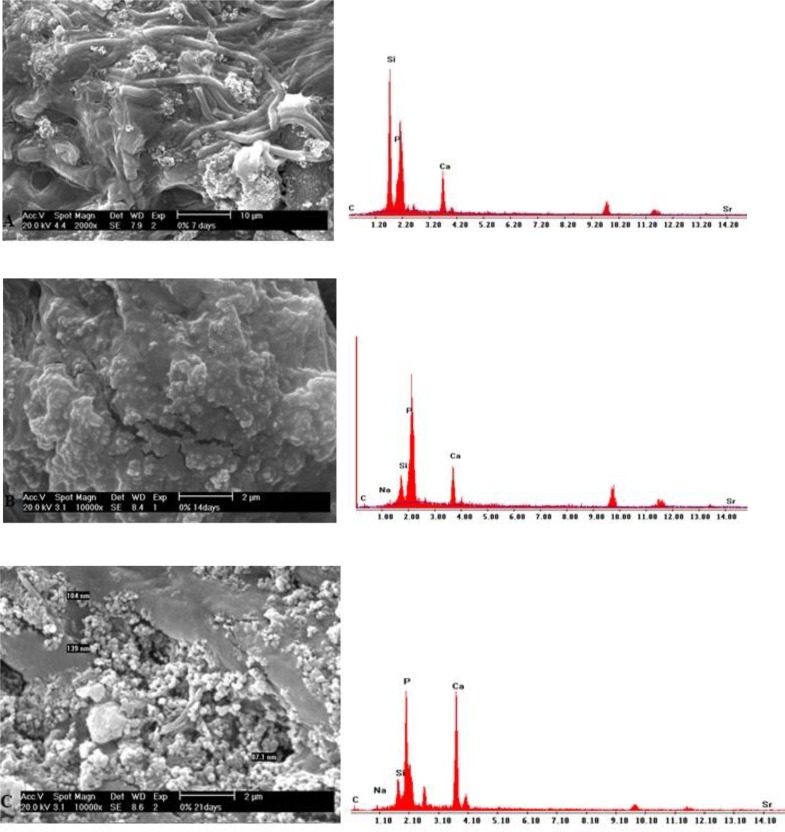
(A) SEM and EDX of BG at day 7. (B) SEM and EDX of BG at day 14. (C) SEM and EDX of BG at day 21

**Fig. 4:**
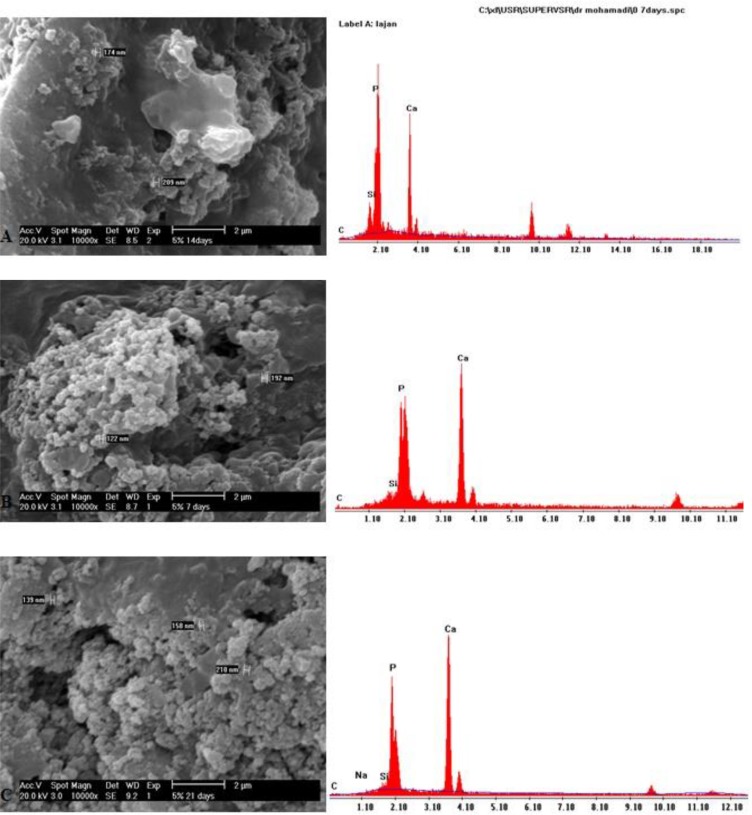
(A) SEM and EDX of BG modified with 5% Sr at day 7. (B) SEM and EDX of BG modified with 5% Sr at day 14. (C) SEM and EDX of BG modified with 5% Sr at day 21

**Fig. 5:**
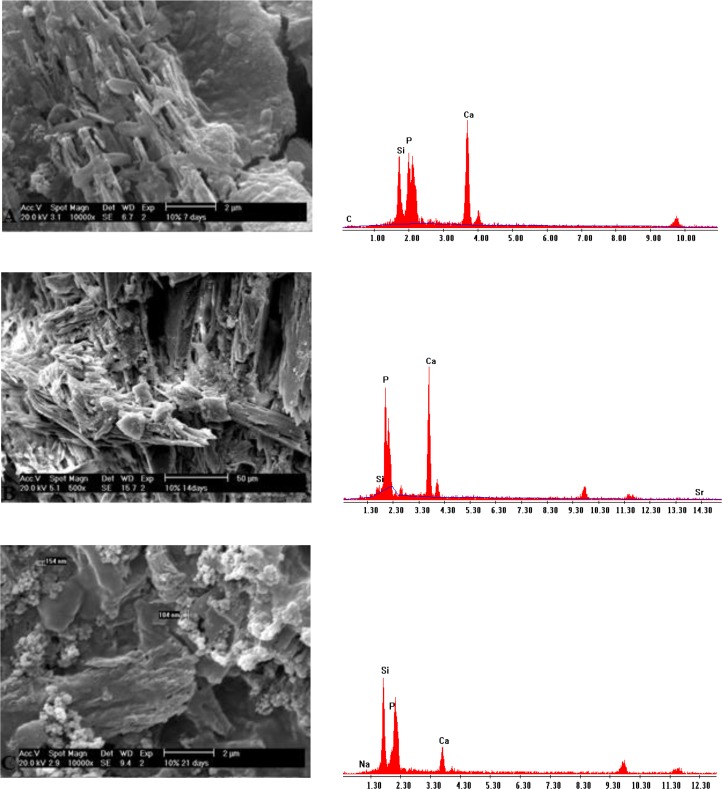
(A) SEM and EDX of BG modified with 10% Sr at day 7. (B) SEM and EDX of BG modified with 10% Sr at day 14. (C) SEM and EDX of BG modified with 10% Sr at day 21

SEM micrographs of the BG group at day 7 showed globular hydroxyapatite crystals on the dentin surface. At days 14 and 21, the developed apatite was more homogeneous and greater in the amount in comparison with day 7 (Fig. 3: A–C). In the group of BG modified with 5% Sr, SEM micrographs demonstrated the gradual growth of globular hydroxyapatite crystals (Fig. 4: A–C). SEM micrographs of the group of BG modified with 10% Sr at days 7 and 14 showed more hydroxyapatite crystals in comparison to day 21. These hydroxyapatite crystals were needle-shaped and distinct; however, hydroxyapatite formation decreased, and the crystals became less distinct at day 21 (Fig. 5: A–C).

### SEM analysis of tubular obstruction:

**Fig. 6:**
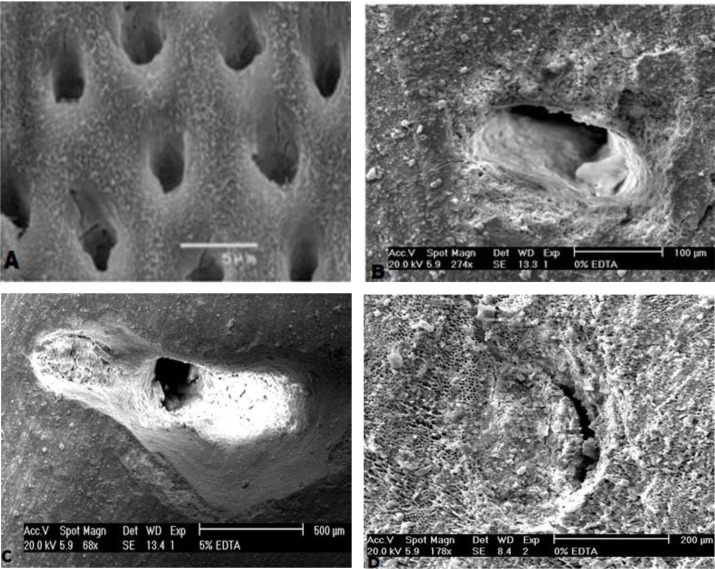
**(**A) SEM of dentin discs after etching and before treatment. (B) SEM of BG, showing the partial tubular obstruction. (C) SEM of BG modified with 5% Sr, demonstrating partial tubular obstruction. (D) SEM of BG modified with 10% Sr, exhibiting complete tubular obstruction

After etching the dentin discs, open dentinal tubules were clearly visible under SEM. The SEM images of the dentin discs demonstrated layers of BG particles on the dentin surface, and dentinal tubules were partially occluded by BG and BG modified with 5% Sr, while they were almost completely occluded by BG modified with 10% Sr (Fig. 6: A–D).

## DISCUSSION

BG, a highly biocompatible calcium sodium phosphosilicate compound, is used in the dental setting for air-polishing procedures. Moreover, it is added to the formulation of bonding agents, varnishes, toothpastes and desensitizing pastes, in order to create hybrid remineralizing agents [16,17].

BG may react with saliva and may induce the dissolution of Ca^2+^, PO_4_^3−^ and Si^4+^ ions from the glass surface, resulting in subsequent precipitation of a polycondensed silica-rich layer (Si-gel), which acts as a matrix for the formation of calcium phosphate (CaP), subsequently crystallizing into HCA [18]. Thus, BG acts as a remineralizing agent in non-cavitated lesions, or in patients at high risk of developing caries. The BG used in this study formed HCA in demineralized dentin. The remineralization process induced by BG in this study was most likely due to a simultaneous bioactive phenomenon, which is characterized by the release of Si^4+^ ion and a subsequent polycondensation reaction, which is induced by deposition of calcium and phosphate ions on the organic matrix and formation of a calcium phosphate phase. The polycondensation reaction forms H_2_O, which physically bonds to the Si–O–Si surface and forms a hydrated silica-rich layer [19, 20]. As a result of incorporation of soluble Ca^2+^ and PO_4_^3^ ions, a polycondensed silica-rich layer (Si-gel) is then deposited in the form of an amorphous CaO-P_2_O_5_. Following the uptake of mineral ions from artificial saliva, the amorphous CaO-P_2_O_5_ is converted to HCA [21]. In the current study, high calcium and phosphate peaks on EDX, and the increased mineral matrix area ratio in ATR-FTIR and XRD analyses supported the above-mentioned processes. The pH cycling which was used in this study for creating caries-like lesions has also been used in a previous study [22]. In the current study, BG modified with 10% Sr showed more obvious remineralization at days 7 and 14; however, the remineralization decreased at day 21. BG modified with 5% Sr exhibited an increase in remineralization over time, similar to BG.

Spectroscopic analyses by FTIR have been commonly used to qualitatively confirm the formation of apatite in studies focusing on dentin remineralization. Spectroscopy is a non-destructive approach enabling continuous assessment of remineralization processes [23]. By only accepting powder samples, ATR-FTIR was shown to be an effective method for detecting dentin minerals. ATR-FTIR spectroscopy is a commonly used non-destructive approach for characterizing chemical changes in mineralized tissues. ATR-FTIR spectroscopy showed that remineralization in BG group increased at days 7, 14 and 21. Wang et al [9] evaluated dentin remineralization after 7 days of immersion in artificial saliva and assessed the induction of remineralization using 45S5 BG and modified BG (soda-lime spherical glass). The authors showed that both agents remineralized the partially and totally demineralized dentin. The remineralizing effect on completely demineralized dentin was less obvious when compared to partially demineralized dentin, which is possibly due to the difficulty in crystal nucleation [9]. Vollenweider et al [24] evaluated the remineralizing effect of 45S5 nano-BG on dentin for 30 days and reported the highest remineralization rate at day 30, which was in line with the current findings. Prabhakar et al [25] reported that 45S5 BG had a remineralizing effect on demineralized enamel after 30 days. Additionally, Narayana et al [2] evaluated the efficacy of BG for remineralization of artificial carious enamel lesions after 10 days. The authors found BG to be an effective remineralizing agent [2]. The results of the aforementioned studies are in accordance with the findings of the current study. Sr ions stimulate bone formation by osteoblasts and prevent osteoclastic bone resorption [26].

Sr-substituted BG is suitable for bone repair and regeneration, and it is a promising bone substitute material. Sr-releasing BG is a wise choice in orthopedic applications, tissue engineering, and coating of metallic implants [27]. BG is also added to toothpaste as a remineralizing agent, especially for treating DH by precipitating HCA on the tooth surface and dentinal tubules [28, 29]. The addition of Sr to BG is beneficial because Sr has been shown to prevent dental caries while improving enamel remineralization [11]. Modified BG seems to be a suitable choice when considering its positive effects on apatite and the potential for a more controlled release of Sr ions for treatment of DH and prevention of caries [11].

Issac et al [30] found that 5% Sr releases ions and forms apatite. Therefore, the current study used 5% and 10% concentrations of Sr. In the ATR-FTIR analysis, a change at the 1030 cm^−1^ peak was detected as the percentage of Sr increased, indicating that Sr has induced glass breakdown and hydroxyapatite formation; thus, Sr can increase remineralization. ATR-FTIR spectroscopy showed that in the group of BG modified with 10% Sr, the intensity of this peak decreased at day 21 when compared to days 7 and 14, which indicates the increased solubility of HCA crystals. The intensity of the apatite peak in the group of BG modified with 5% Sr at day 21 was higher than that in other groups; indicating that 5% Sr not only increases remineralization but also stabilizes the apatite lattice. Shahid et al [31] indicated that replacement of calcium by Sr in glass-ionomer decreased the solubility of apatite during acid attacks. In XRD, BG modified with 5% Sr exhibited an increase at 2θ=26° and 2θ=32°–34° peaks, and the peaks at day 21 were greater than those at days 7 and 14; which means that 5% Sr has stabilized the apatite lattice. Issac et al [30] found that 5% Sr caused bone formation, which was in line with the current findings. The XRD of the dentin treated with BG modified with 10% Sr at days 7 and 14 showed sharp peaks with high intensity at 2θ=26° and 2θ=32–34°, which represented the greater formation of apatite. At day 21 of remineralization, these peaks significantly decreased due to apatite dissolution. Increasing Sr in the apatite lattice prevented the growth of apatite sheets and disturbed apatite organization. Gentleman et al [12] assessed the effect of substitution of calcium with Sr in BG on apatite formation at the percentages of 0, 2.5, 10, 50 and 100%, and showed that bone remineralization increased by increasing the percentage of Sr; however in that study, the samples were evaluated after 480 minutes, and remineralization was not assessed at longer periods of time. Moreover, bone needs more Sr for remineralization in comparison with dentin. DH is related to the movement of fluids within dentinal tubules. According to the Poiseuille’s law, this movement of fluid is “directly proportional to the fourth power of the radius”; therefore, reduction in the radius of the tubule would be anticipated to decrease dentin permeability, which should be effective in the treatment of DH [14]. SEM images of the dentin discs with open tubules demonstrate layers of BG particles on the dentin surface and show occlusion of dentinal tubules by means of crystal-like apatite.

According to SEM imaging, the use of all the three compositions of BG led to occlusion of open tubules, as BG and BG modified with 5% Sr partially occluded the tubules, and BG modified with 10% Sr completely sealed the tubules. Lynch et al [14], and Ramamoorthi and Nivedhitha [10] showed that BG may decrease DH. In the study by Gupta et al [32], BG completely occluded the dentinal tubules, while Gluma desensitizer caused partial occlusion.

## CONCLUSION

Within the limitations of this study, it may be concluded that the two formulations of Sr-containing BG have a high remineralization potential, as confirmed by SEM, XRD and ATR-FTIR analyses. Addition of 5% Sr stabilizes the apatite lattice and exhibits resistance against dissolution. All BG compositions used in the present study sealed the dentinal tubules; however, BG modified with 10% Sr resulted in a complete tubular occlusion.
